# Circular RNAs and RNA Splice Variants as Biomarkers for Prognosis and Therapeutic Response in the Liquid Biopsies of Lung Cancer Patients

**DOI:** 10.3389/fgene.2019.00390

**Published:** 2019-05-07

**Authors:** Florence de Fraipont, Sylvie Gazzeri, William C. Cho, Beatrice Eymin

**Affiliations:** ^1^INSERM U1209, CNRS UMR5309, Institute for Advanced Biosciences, University Grenoble Alpes, Grenoble, France; ^2^Grenoble Hospital, La Tronche, France; ^3^Department of Clinical Oncology, Queen Elizabeth Hospital, Kowloon, Hong Kong

**Keywords:** cancer biomarkers, circular RNAs, liquid biopsies, lung cancer, RNA splice variants

## Abstract

Lung cancer, including non-small cell lung carcinoma (NSCLC), is the most frequently diagnosed cancer. It is also the leading cause of cancer-related mortality worldwide because of its late diagnosis and its resistance to therapies. Therefore, the identification of biomarkers for early diagnosis, prognosis, and monitoring of therapeutic response is urgently needed. Liquid biopsies, especially blood, are considered as promising tools to detect and quantify circulating cancer biomarkers. Cell-free circulating tumor DNA has been extensively studied. Recently, the possibility to detect and quantify RNAs in tumor biopsies, notably circulating cell-free RNAs, has gained great attention. RNA alternative splicing contributes to the proteome diversity through the biogenesis of several mRNA splice variants from the same pre-mRNA. Circular RNA (circRNA) is a new class of RNAs resulting from pre-mRNA back splicing. Owing to the development of high-throughput transcriptomic analyses, numerous RNA splice variants and, more recently, circRNAs have been identified and found to be differentially expressed in tumor patients compared to healthy controls. The contribution of some of these RNA splice variants and circRNAs to tumor progression, dissemination, or drug response has been clearly demonstrated in preclinical models. In this review, we discuss the potential of circRNAs and mRNA splice variants as candidate biomarkers for the prognosis and the therapeutic response of NSCLC in liquid biopsies.

## Introduction

Lung cancer is the most common cause of cancer-related deaths (Ferlay et al., 2015; Siegel et al., 2017). Non-small cell lung cancer (NSCLC) comprises mainly lung adenocarcinoma, squamous cell lung carcinoma, and large cell lung carcinoma. It represents about 85% of total lung cancer patients. Over the past decades, the emergence of therapies [such as those targeting the epidermal growth factor receptor (EGFR), the anaplastic lymphoma kinase (ALK), or the immune checkpoints programmed cell death (PD-1)/programmed death ligand (PD-L1)] has led to an unprecedented improvement of progression-free survival in a subset of advanced NSCLC patients. However, the long-term survival for NSCLC patients remains very poor and is still closely related to the stage of the disease (Chansky et al., 2017; Kris et al., 2017). This poor prognosis is mainly due to the intrinsic aggressiveness of NSCLC, the late diagnosis of the disease, and the recurrent development of resistance to targeted therapies. Therefore, the identification of biomarkers for early detection and selection of high-risk subjects as well as the characterization of predictive biomarkers (at both baseline and progression) of therapeutic response are urgently needed. This could improve the clinical management of these patients and help to understand the tumor progression mechanisms under treatment. In this context, less-invasive, specific, reproducible, and sensitive pre-analytical and analytical methods for the collection and analysis of tumor samples have to be developed, improved, and standardized.

Circular RNAs (circRNAs) are generated by back splicing. Unlike linear RNAs, they have closed-loop structures coming from the ligation of exons, introns, or both (Dragomir and Calin, 2018; Wang et al., 2019). Although first described in the early 1970s, circRNAs were until very recently considered as the by-products of splicing without any important biological functions. These recent years, the development of next-generation sequencing technologies together with the improvement of bioinformatic pipelines has allowed the identification of numerous circRNAs that are differentially expressed (either up- or down-regulated) in cancer patients compared to normal controls (Dragomir and Calin, 2018). Therefore, a new area of research is now emerging to investigate the role of circRNAs as candidate biomarkers for the diagnosis or prognosis of cancer.

In eukaryotes, gene expression is finely controlled by complex regulatory processes that affect all steps of RNA expression, extending from transcription to maturation in the nucleus, mRNA cytosolic export, stability, and translation. One of the crucial steps is the pre-mRNA constitutive splicing process, which removes intronic sequences from the pre-mRNA and joins exonic sequences to form the mature mRNA. Another level of regulation is represented by pre-mRNA alternative splicing (Stamm et al., 2005; Hallegger et al., 2010). Through the use of alternative promoters, alternative polyadenylation sites, intron retention, or alternatively spliced exons (exon cassette or mutually exclusive exon), this process generates several coding mRNA splice variants from the same gene. Therefore, one of the main consequences of alternative splicing is to remodel the proteome through the synthesis of various protein isoforms with different biological activities. This process is tightly controlled across different tissues and developmental stages, and the dysregulation of alternative splicing has been closely associated with various human diseases including cancer (Pan et al., 2008; Wang E.T. et al., 2008). Thus, a deepened characterization of pre-mRNA processing events that are deregulated in tumors is required to uncover new oncogenic mechanisms as well as to design alternative therapeutic strategies.

In this review, we discuss the importance of circRNAs and RNA splice variants as cancer biomarkers, with a focus on lung cancer. We also discuss the pros and cons of RNA liquid biopsies as promising but challenging approaches to identify potential RNA biomarkers in cancer.

## Rna Liquid Biopsies: Promising Tools for the Detection of Cancer Biomarkers?

In clinical practice, repeated tissue biopsies can be used to monitor tumor evolution. However, these are invasive procedures with complication risks for patients. In addition, single-site tissue biopsy represents a snapshot of the tumor, often provides insufficient materials, and is subjected to selection bias resulting from tumor heterogeneity. Tumor liquid biopsies can be considered as a non-invasive surrogate of tissue biopsies. Furthermore, circulating markers more likely reflect systemic tumor burden and depict intratumoral heterogeneity that occurs in metastatic lesions (Cai et al., 2015; Siravegna et al., 2017; Rolfo et al., 2018). Liquid biopsies are therefore a highly relevant alternative to tissue biopsies for initial molecular diagnosis and management of tumor progression, especially following treatment.

Beside proteins, various tumor-derived components can be isolated from blood including circulating cell-free tumor DNA (ctDNA), cell-free tumor RNA (ctRNA), circulating exosomes, tumor educated platelets (TEPs), and circulating tumor cells (CTCs) (Joosse and Pantel, 2015; Reclusa et al., 2017; Raez et al., 2018). It was previously found that tumor patients have higher levels of ctDNA compared to healthy donors, and metastatic patients even more than non-metastatic patients (Leon et al., 1977). In NSCLC patients, ctDNA sequencing of tumor plasma was reported to be as useful as tumor tissue genotyping to follow the appearance of specific mutations such as EGFR(T790M) mutations at the time of tumor progression (Oxnard et al., 2016). More recently, Abbosh et al. (2017) identified differential phylogenetic ctDNA profiles associated with adjuvant chemotherapy resistance and lung cancer relapse and metastasis. Therefore, detection of ctDNA in the plasma could also be used to track the appearance/selection of lung cancer subclones during progression upon treatment.

Circulating RNAs are also detected in tumor liquid biopsies. As an example, it has been shown that platelets sequester tumor-related RNAs by a microvesicle-dependent mechanism, resulting in the generation of TEPs (Best et al., 2015). Importantly, RNA sequencing of platelets from 60 patients with NSCLC (56 metastatic) and 55 healthy donors identified a specific tumor signature (Best et al., 2015). Plasma cell-free RNA has also been investigated for a long time (Kopreski et al., 1999) and used for the measurement of RNA transcripts of fusion genes (SLIT, ALK, ROS1, and RET) by reverse transcription polymerase chain reaction (RT-PCR) in lung cancer (Zhao et al., 2014). New gene fusions such as those leading to the constitutive activation of the tropomyosin receptor kinase TrkA encoded by the NTRK1 gene have been identified in lung cancer patients (Vaishnavi et al., 2013; Amatu et al., 2016). As the TrkA inhibitor larotrectinib was very recently approved by the FDA for patients with advanced solid tumors carrying NTRK gene fusions, the detection of these transcripts in plasma could be a valuable tool for the management of these patients. Circulating RNAs have substantial advantages over ctDNA: (1) RNA expression level is highly dynamic and fluctuates according to the proliferative state or metabolism of cancer cells; (2) the expression pattern of RNA molecules, including splice variants or long non-coding RNAs (lncRNAs), is highly specific of tissue types or disease stage; and (3) focusing on RNA provides a combined analysis of a multitude of RNAs (lncRNAs, miRNAs, piwi-interacting RNAs, fusion transcripts, splice variants, or RNA modifications). These last years, owing to the development of high-throughput sequencing technologies, the number of RNAs belonging to each category has extensively increased, providing an unlimited reservoir of potential cancer biomarkers in various cancer types (Nigita et al., 2018; Obermannova et al., 2018). Conversely, circulating RNAs have substantial disadvantages over ctDNA: (1) circulating free RNA is not stable; (2) RNA transcription varies between people according to different variables such as gender, diet, and age; and (3) different factors such as time of blood draw, blood collection tube, blood fraction (i.e., platelet-rich versus platelet-free plasma), complete blood count, or RNA isolation protocols could influence RNA abundance levels in body fluids, both inside and outside extracellular vesicles. Therefore, carefully annotated health control libraries as well as well-standardized protocols for sample collection and analysis are needed before studying complex diseases such as cancer (Yuan et al., 2016).

## Circrnas: a Promising Source of Biomarkers in Lung Cancer?

### CircRNAs: General Considerations

CircRNAs are generated from linear pre-messenger RNAs through a process termed “back splicing,” in which the 3’ and 5’ ends are joined together, leading to a covalently uninterrupted loop. Although circRNAss were first discovered in the 1970s, they were considered for a long time as misspliced by-products without any important biological roles. In 2012, numerous circular transcripts were discovered and annotated in mammalian cells (Salzman et al., 2012). CircRNAs originate from several types of RNA molecules including exons, introns, long non-coding RNAs, antisense transcripts, or intergenic regions (Dragomir and Calin, 2018). In addition, circRNAs originating from coding regions can contain multiple exons and sometimes also include intronic regions (Maass et al., 2017). This could be dependent on the average length of the pre-mRNA exons and of the flanking introns. Some circRNAs such as Circ-ZNF609 are translated with a role in myogenesis in that case (Legnini et al., 2017). Interestingly, some circRNAs are more abundantly expressed than their associated linear mRNAs (Salzman et al., 2013; Maass et al., 2017). Thus, circRNA formation could result from distinct post-transcriptional regulation by competing with the formation of linear mRNA from the same pre-mRNA (Ashwal-Fluss et al., 2014).

Briefly, three main mechanisms lead to back splicing (Dragomir and Calin, 2018). The first mechanism is defined by exon skipping and intron lariat building, which brings distant exons together and induces the circularization of skipped exons. As a consequence, three different mRNA molecules are formed: an intron lariat, a circRNA, and a linear mRNA with skipped exons (Zaphiropoulos, 1997; Jeck et al., 2013; Kelly et al., 2015). The second mechanism comes from the circularization of exon/exon by the complementary pairing of the flanking introns (Dubin et al., 1995; Liang and Wilusz, 2014; Zhang et al., 2014). Introns that paired often contain inverted ALU repeats (Jeck et al., 2013). The third mechanism is controlled by RNA binding proteins (RBPs) such as quaking (QKI) and musclebind (MBL) proteins, which bind the neighboring introns of the future circular exons and dimerize, creating an RNA loop (Ashwal-Fluss et al., 2014; Conn et al., 2015).

The biological functions of circRNA are scarce (Hentze and Preiss, 2013). Several studies have shown that most of circRNAs contain some, although not multiple, miRNA response element (MRE) (Guo et al., 2014). As a consequence, the role of circRNAs as miR sponges has been extensively investigated and has been reported as an indirect mechanism to increase the expression of numerous mRNAs (Hansen et al., 2013; Memczak et al., 2013; Tay et al., 2014). The majority of circRNAs does not associate with ribosomes or the translational machinery (Guo et al., 2014). However, in some cases, the presence of an internal ribosome entry site (IRES) in the circRNA sequence would allow translation, at least *in vitro* (Chen and Sarnow, 1995). In agreement with these results, it was reported that both circZNF609 and circMBL associate with polysomes (at low levels) and are translated into short proteins, which contain a functional domain (Legnini et al., 2017; Pamudurti et al., 2017). The exact function of these peptides is yet unknown. Similar to miRNA sponging, circRNAs also bind RBPs such as MBL (Ashwal-Fluss et al., 2014), SR proteins (SRSF1), (Barbagallo et al., 2018), or IGF2BP3 (Schneider et al., 2016). Again, the functional consequences of these interactions remain elusive and controversial. Different explanations have been proposed: (1) circRNAs could be vehicles for RBPs, allowing their delivery in a specific subcellular localization; (2) circRNAs could be sponges for RBPs and could inhibit their function by sequestration; (3) circRNAs could act as a platform for multiple RBPs, allowing their interaction; and (4) circRNAs could bind RBPs and induce allosteric changes that regulate their functions.

Up to now, the signaling pathways that control the formation of circRNA loops remain largely unknown. Notably, the mechanisms that deregulate circRNA expression in cancers are unknown. In Drosophila, it was found that SR (serine rich) and hnRNP proteins, which are crucial regulators of both constitutive and alternative splicing, act in a combinatorial manner to regulate back splicing of many pre-mRNAs. This suggests that the spliceosome machinery activity controls the balance between linear and circular mRNAs (Kramer et al., 2015). Consistent with this idea, depletion or pharmacological inhibition of the spliceosome components of the SF3b or SF3a complexes, which include SR proteins or hnRNPs, increases the steady-state levels of circRNAss while concomitantly decreases the linear mRNAs (Liang et al., 2017). These results demonstrate that inhibition or slow-down of canonical pre-mRNA processing events shifts the steady-state output of protein-coding genes toward circRNAss. Interestingly, mutations in spliceosome genes such as SF3B1, SRSF2, and U2AF1 are prevalent in several cancers, including lung cancers (Chabot and Shkreta, 2016). Therefore, it is tempting to speculate that these mutations as well as other molecular alterations (amplification, translocation…) of specific cancer-related genes may affect circRNA biogenesis and contribute to disease progression. Although this hypothesis has not been validated yet, a link between circRNA expression, KRAS mutation, and c-MYC overexpression has been reported in lung and colorectal cancers (Dou et al., 2016; Gou et al., 2017).

### CircRNAs as Prognostic Biomarkers in Lung Cancer

Many studies have analyzed circRNA expression in lung cancer cell lines (Panda et al., 2017; Tian et al., 2017), as well as in tissue samples and liquid biopsies (plasma) from lung cancer patients (Hu et al., 2018; Ma Y. et al., 2018; Wang et al., 2019). Up to now and based on PubMed database, about 50 papers have identified circRNAs in lung cancer. As most of these papers were published last year, it is obvious that the number of studies will exponentially increase in the future. In 2018, Ding et al. (2018) performed a genome-wide transcriptome profiling of circRNA in paired lung adenocarcinoma and healthy lung tissues using ribosomal RNA-depleted RNA sequencing. They identified 9,340 circRNA candidates. Although circRNAs were often weakly expressed and identified on less than 10 reads for more than half of them, these results are consistent with their wide expression in lung adenocarcinoma. Supplementary Tables 1, 2 list circRNAs that are either up-regulated (Supplementary Table 1) or down-regulated (Supplementary Table 2) in lung cancer compared to normal lung. Below are examples of these circRNAs. Some of them have been proposed as potential diagnostic and/or prognostic biomarkers. Mechanistically, most of these circRNAs act as miR sponges.

Many circRNAs are down-regulated and are thus considered as tumor suppressors. Down-regulation of the circRNA transcript of the tumor suppressor gene ITCH was reported in a large series of lung cancer patients (Wan et al., 2016). Mechanistically, circ-ITCH sponged miR-7 and mir-214. Its down-regulation induced the activation of the Wnt/beta-catenin signaling pathway through the up-regulation of T-cell factor, beta-catenin, c-Myc, and cyclin D1 (Wan et al., 2016). The down-regulation of various circRNAs targeting ZEB1 (circ-ZEB1.5, circ_ZEB1.19, circ_ZEB1.17, and circ_ZEB1.33) increased the level of miR-200, which promoted tumor lung metastasis (Liu et al., 2016; Wang F. et al., 2016). Expression of circPTK2 was reduced in NSCLC cells. This activated TGF-beta-induced epithelial to mesenchymal transition and invasion through a miR-429/miR-200b-3p axis and an increased expression of SNAIL (Wang L. et al., 2018). Decreased expression of circ-FOXO3 was also found in lung cancer (Zhang Y. et al., 2018). As circ-FOXO3 binds focal adhesion kinase (FAK) and hypoxia inducible factor 1 (HIF1) alpha proteins and prevents their nuclear translocation (Du et al., 2017), it was assumed that its down-regulation enhanced the angiogenic and invasive properties of FAK and HIF1. Finally, the low expression of Hsa_circ_0033155 (Gu et al., 2018) and hsa_circ_100395 (Chen et al., 2018) was associated with a poor prognosis in NSCLC patients.

Conversely, many circRNAs are up-regulated in lung cancer and are thus considered as tumor promoters. circ-404833 and circ-406483 are highly expressed in the tissues of early-stage lung cancer patients compared to control tissues (Zhao et al., 2017). Up-regulation of hsa_circ_0012673 was observed in lung adenocarcinoma tumor samples when compared to pair-matched adjacent non-tumoral tissues and was associated with tumor size (Wang X. et al., 2018). Moreover, expression level of numerous circRNA, such as hsa_circ_0013958 (Zhu et al., 2017), hsa_circ_0000064 (Liu et al., 2018; Zhang S. et al., 2018), ciRS-7 (Yan et al., 2018), circRNA_102231 (Zong et al., 2018), hsa_circRNA_103809 (Liu et al., 2018), circFADS2 (Zhao et al., 2018), circ_0067934 (Wang and Li, 2018), hsa_circ_0000729 (Li S. et al., 2018a), circ-BANP (Han et al., 2018), circ_0016760 (Li Y. et al., 2018), hsa_circ_0020123 (Qu et al., 2018), and circPRKCI (Qiu M. et al., 2018), was associated with TNM stage and lymphatic metastasis in lung cancer patients. These cirRNAs have been proposed as potential biomarkers for the early detection and screening of lung cancer. Mechanistically, *in vitro* studies also demonstrated that circRNAs promote cancer cell proliferation, migration, and invasion as well as prevent apoptosis by acting as miR sponges that regulate gene expression. As an example, hsa_circ_0013958 promoted cancer cell proliferation and invasion by acting as a miR-34 sponge to up-regulate cyclin D1 expression (Zhu et al., 2017). Circ-MAN2B2 promoted the expression of FOXK1 by sponging miR-1275, leading to cell proliferation and invasion (Ma X. et al., 2018). Expression of Hsa_circ_0007385 was associated with cell proliferation, invasion, and metastasis, which could be related with its sponge activity against miR-181 (Jiang et al., 2018). In A549 and H1299 lung adenocarcinoma cells, hsa_circ_0000064 promoted cell proliferation, migration, and invasion and prevented apoptosis (Luo et al., 2017). Some of its targets included cell cycle regulators (p21^WAF1^, CDK6, and cyclin D1) and apoptotic factors (caspase-3, caspase-9, and bax). Hsa_circ_0012673, a known miR-22 sponge, enhanced the proliferation of lung adenocarcinoma cells through up-regulation of ErbB3 expression (Wang X. et al., 2018). As circRNA_100876 is a sponge for miR-136 that targets the matrix protease MMP13, increased MMP13 activity may explain the association between up-regulation of circRNA_100876 and lung tumor progression (Yao et al., 2017). Circ-FGFR3 activated a galectin-1/AKT/ERK signaling pathway by sponging miR-22-3p, and promoted cell invasion and proliferation (Qiu B.Q. et al., 2018). Very recently, Li L. et al. (2018) identified FECR1, an exonic circRNA resulting from the back splicing of exon 4-2-3 of the friend leukemia virus integration 1 (FLI1) transcript, as a driver oncogene that promoted tumor metastasis in small cell lung carcinoma (SCLC). They showed that FECR1 sponges miR584-3p and induces the activation of the rho-associated coiled-coil kinase 1 (ROCK1), which promotes metastasis. Interestingly, Qiu M. et al. (2018) found that amplicon 3q26.2, which is highly susceptible to gene mutation in lung cancer, produces circPRKCI and promotes cell proliferation and migration through a circPRKCI-miR545/589-E2F7 axis.

As a whole, these studies clearly indicated that circRNAs contribute to the progression of lung cancer, especially NSCLC.

### Circulars RNAs and Lung Cancer Response to Therapies

Although targeted therapies and immunotherapies have revolutionized the therapeutic management of NSCLC patients, resistance develops. Therefore, further research is needed to identify predictive biomarkers for the follow-up of patients. To our knowledge, only very few studies have investigated the regulation of circRNAs (biogenesis, localization, and functions) in response to treatment. Given the role of circRNAs in lung tumor progression, this is an important topic, although it remains challenging. Moreover, as the access to tumor biopsies before/after relapse remains difficult, the detection of circRNAs in liquid biopsies is a promising alternative for the management of post-treatment follow-up.

Zhou et al. (2019) recently showed that hsa_circ_0004015 increases the resistance of HCC827 NSCLC cell line to gefitinib, an EGFR-tyrosine kinase inhibitor currently used in clinic. Through its ability to sponge miR-1183, hsa_circ_0004015 induced the expression of the 3-phosphoinositide-dependent protein kinase 1 (PDPK) and stimulated AKT pathway (Zhou et al., 2019). Using a high-throughput circRNA microarray, Xu et al. (2018) found a significant up-regulation of 2,909 circRNAs and down-regulation of 8,372 circRNAs in taxol-resistant A549 lung adenocarcinoma cells compared to parental cells. Interestingly, they highlighted circRNA/miRNA networks, suggesting that circRNAs also acted through miRNAs sponging in this context. They found that the most significantly enriched pathways for aberrant circRNA-related host genes include integrin, EGFR, VEGFR, and rho GTPase signaling or degradation of the extracellular matrix, which are all involved in the progression of cancers following chemotherapeutic treatment. Altogether, these studies strongly suggested that dysregulated circRNA expression contributes to the resistance of NSCLC tumors to targeted therapies as well as to chemotherapies.

Another role of circRNAs during cancer growth is the formation of fusion circRNAs (f-circRNAs) produced by chromosomal translocations. These fusion circRNAs were first identified in leukemias (Guarnerio et al., 2016). The EML4-ALK fusion is the result of a chromosomal rearrangement between the N-terminal portion of EML4 and the tyrosine kinase domain of ALK. ALK rearrangements are more prevalent in younger NSCLC patients, females, and never or former light smokers (Shaw et al., 2011). Importantly, EML4-ALK-positive patients derive clinical benefits from ALK tyrosine kinase inhibitors such as crizotinib (Shaw et al., 2013). However, despite initial good responses, tumor progression is systematically observed due to the emergence of resistance mechanisms (Katayama et al., 2012). Interestingly, Rosell’s group detected EML4-ALK rearrangements in RNA isolated from platelets (Nilsson et al., 2016). During the course of the disease, the presence of EML4-ALK fusion transcripts in platelets correlated with the clinical outcome in response to crizotinib treatment. The same group also demonstrated the presence of EML4-ALK fusion transcripts in the plasma by using RT-PCR. Moreover, a F-circRNA derived from back splicing of the EML4-ALK fusion gene (F-circEA-2a) was recently identified (Tan et al., 2018). F-circEA-2a promoted cell invasion and migration but not cell proliferation and colony formation in NSCLC (Tan et al., 2018). F-circEA-2a was detected in tumor tissues but not in the plasma of EML4-ALK-positive patients. It remains to be determined whether F-circEA-2a expression varies upon treatment with crizotinib and whether F-circEA-2a could represent a new predictive RNA biomarker of response to targeted therapies.

### CircRNAs as Valuable Biomarkers in Liquid Biopsies: Pros and Cons

The features of a qualified biomarker include stability, sensitivity, specificity, accuracy, and reproducibility (Henry and Hayes, 2012). CircRNAs encompass some of these features. Due to the covalently closed-loop structure lacking free 5’ and 3’ ends, circRNA molecules are highly resistant to exonuclease RNAse R (Jeck et al., 2013) and are thus much more stable than linear RNAs (Memczak et al., 2013; Enuka et al., 2016). Hence, the average half-life of circRNAs in plasma exceeds 48 h, while that of mRNAs is 10 h on average (Jeck and Sharpless, 2014). In addition, circRNAs are tissue and developmental stage specific. They are differentially expressed between cancerous and non-cancerous tissues (Memczak et al., 2013). Their differential pattern of expression allows to distinguish cancer types and/or histological subtypes (Salzman et al., 2013). Therefore, owing to these general features, circRNAs have been considered as highly suitable biomarkers for liquid biopsies. circRNAs are detectable in body fluids such as saliva (Bahn et al., 2015) or whole blood (Memczak et al., 2015), and are also abundant in exosomes (Li et al., 2015). circRNAs are detected in the free-floating cells inside body fluids such as circulating blood cells (platelets and erythrocytes) and CTCs. Maass et al. (2017) found that platelets exhibit the highest expression of circRNAs among 20 different tissues tested. Although the expression of circRNAs is abundant in the plasma/serum, many of these circulating circRNAs do not discriminate between cancer and non-cancer patients (Henry and Hayes, 2012). Thus, only a few circRNAs could be differentially expressed in cancerous patients liquid biopsies. As an example, Li L. et al. (2018) recently detected FECR1 circRNA in exosomes purified from SCLC patient sera. The expression of serum-exo-FECR1 was aberrantly increased in SCLC patients compared with normal healthy donors and was associated with disease progression following chemotherapy (Li L. et al., 2018).

Therefore, before using circRNAs as cancer biomarkers in liquid biopsies, we have to carefully decipher the biological material that is the most relevant for detection (plasma, serum, whole blood, and platelets) and to better characterize circRNA expression level in relevant disease controls (healthy donors) and relevant control cells. We also have to optimize and standardize the pre-analytical methods of circRNA purification as well as the analytical procedures and bioinformatic pipelines.

## Rna Splice Variants asBiomarkers of CancerProgression and Response toTherapies in Lung Cancer

### Alternative Splicing and Cancer Progression

**FIGURE 1:**
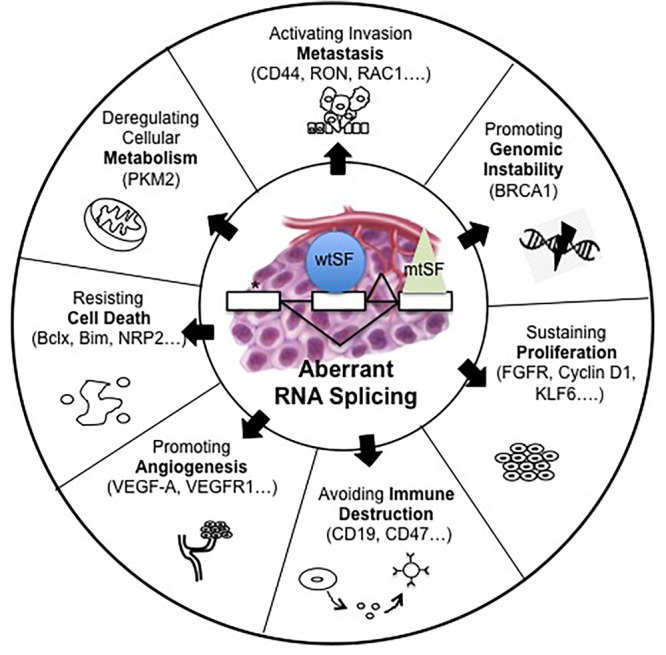
Alterations of pre-mRNA alternative splicing in cancer. Human tumors display mutations in *cis*, which alter splice sites (^∗^) or abnormalities in *trans* such as somatic mutations (mtSF) or deregulated expression of splicing regulatory factors (wtSF). These alterations lead to aberrant RNA splicing of numerous downstream target genes. These abnormal splicing patterns contribute to all cancer hallmarks including genomic instability, sustained proliferation, immune escape, angiogenesis, resistance to cell death, deregulated metabolism, or metastatic dissemination.

It is now well known that abnormal pre-mRNA alternative splicing, through either consensus splice sites mutations or deregulated expression and/or mutations of splicing factors, occurs in cancer and leads to the differential expression of numerous RNA splice variants (Pio and Montuenga, 2009; Syafrizayanti et al., 2018; Figure 1). In this setting, we previously reported the up-regulation of SRSF1 and SRSF2 proteins and SRPK1 and SRPK2 kinases in NSCLC and neuroendocrine lung tumors compared to normal lung tissues (Edmond et al., 2011; Gout et al., 2012). Moreover, and consistent with a role of aberrant splicing in lung tumors, alterations in the splicing pattern of several genes including CD44, Bcl-x, cyclin-D1, FHIT, KLF6, or VEGF-A were associated with lung cancer progression (Pio and Montuenga, 2009; Syafrizayanti et al., 2018; Table 1). Recently, we showed that VEGF_165_b, a splice variant of VEGF-A, regulates a proliferative and invasive autocrine loop in lung adenocarcinoma cell lines and accumulates in lung adenocarcinoma patients, where it correlates with lymph node metastases (Boudria et al., 2018). Interestingly, we detected an increase in the VEGF_165_b protein in the serum of lung adenocarcinoma patients compared to healthy donors. Moreover, we demonstrated that sVEGFR1-i13, a splice variant of the vascular endothelial growth factor receptor 1 (VEGFR1), is up-regulated in squamous lung carcinoma and promotes the proliferation and survival of tumor cells *via* a beta1 integrin-dependent autocrine loop (Abou Faycal et al., 2018). Altogether, these results indicated that abnormal pre-mRNA splicing contributes to the progression of lung tumors.

**Table 1:** Examples of genes with splice variants associated with lung cancer progression and/or response to therapies.

Examples of genes with cancer-related splice variants	Splicing events in tumors	Functional consequences
**INVOLVED IN PROGRESSION/METASTASIS**		
BCL-X	Selection of a distal alternative 5’ splice site	Generation of Bcl-xL, an anti-apoptotic protein overexpressed in NSCLC, which deregulates the balance between pro-apoptotic and anti-apoptotic signals
CD44	Inclusion of various exons (v1–v10)	Generation of various CD44v splice variants including CD44v8-10 with pro-tumorigenic and metastatic functions
CYCLIN D1	Retention of intron 4 due to a G/A870 polymorphism at the exon 4/intron 4 junction	Generation of cyclin D1b, a pro-tumorigenic splice variant
FGFR	Mutually exclusive exon 8 or 9	Generation of distinct extracellular Ig-like domain III with distinct affinity for FGF ligands. Induction of EMT, invasion, and motility
KLF6	Selection of a distal alternative 5’ splice site	Generation of a dominant-negative splice variant termed KLF6-SV1 with a critical role in promoting cell proliferation, survival, and migration
NUMB	Inclusion of exon 9 in tumors	Reduced levels of NUMB protein expression and activation of the pro-tumorigenic NOTCH signaling
MET	Skipping of exon 14	Activation of MET kinase activity Oncogenic transformation
TP53	Selection of an alternative 3’ splice site in intron 6	Generation of a p53 splice variant inducing EMT (epithelial to mesenchymal transition) markers and increasing the motility and invasive properties of lung cancer cells
NRP2	Differential alternative last exon (ALE)	In NSCLC, NRP2b contributes to the oncogenic response to TGFβ and correlates with tumor progression in patients
VEGF-A	Differential exons inclusion/skipping	Generation of various VEGF_xxx_ splice variants with pro-angiogenic activity
VEGFR1	Various intron retention followed by premature polyadenylation	Production of soluble decoy sVEGFR1 acting as negative regulators of VEGFRs signaling on endothelial cells. sVEGFR1-i13 (intron 13 retention) promotes tumor cells proliferation in squamous lung carcinoma
**INVOLVED IN DRUG RESPONSE/RESISTANCE**		
BIM	Skipping of exon 4 due to an intronic deletion polymorphism	Generation of a BIM-γ splice variant lacking BH3 domain BIM-γexpression correlates with resistance to EGFR-TKI in NSCLC cells with EGFR mutation
KLF6	Selection of a distal alternative 5’ splice site	The splice variant KLF6-SV1 promotes resistance of lung adenocarcinoma cells to platinum salts
MET	Skipping of exon 14	Confers sensitivity to MET inhibitors
NRP2	Differential ALE	NRP2b abundance is associated with acquired EGFR inhibitor resistance
VEGF-A	Selection of a distal alternative 3’ splice site in last exon 8	Generation of VEGF_xxx_b splice variants with anti-angiogenic function VEGF_xxx_b are involved in the escape of lung cancer cells from anti-angiogenic therapies
VEGFR1	Intron 13 retention followed by premature polyadenylation	Generation of a sVEGFR1 splice variant involved in the resistance to anti-angiogenic therapies in squamous lung carcinoma

As many studies demonstrate aberrant splicing in cancer, therapeutic strategies to correct these alterations have been developed. Below are some of the strategies that produced very encouraging results in preclinical models of different cancer types, including lung cancer. Importantly, some of these studies unraveled splicing events with a critical role for tumor cell proliferation and/or survival in response to these therapies. Several strategies have been proposed to correct splicing. They include the use of splice-switching oligonucleotides (SSOs), which prevent the binding of splicing factors to their cognate sequence (Bauman et al., 2010). As an example, enhanced exon 6 inclusion leads to the expression of full-length MDM4 in a large number of cancers. The use of antisense oligonucleotide-mediated skipping of exon 6 decreased MDM4 abundance, inhibited tumor growth, and enhanced sensitivity to MAPK-targeting therapies (Dewaele et al., 2016). In addition, redirection of Bcl-x splicing from Bcl-xL (anti-apoptotic isoform) to Bcl-xS (pro-apoptotic isoform) induced apoptosis and increased chemosensitivity in human lung adenocarcinoma cell lines (Taylor et al., 1999). Pharmacological inhibitors targeting different components of the spliceosome machinery, either splicing factors or splicing regulators, have also been developed (Lee and Abdel-Wahab, 2016; Lee et al., 2016). Spliceostatin A, meayamycin B and sudemycins (spliceostatin A analogs), and E7107 (pladienolide analog) bind SF3B1 to prevent the formation of a U2 snRNP-SF3B1 complex with pre-mRNAs (Salton and Misteli, 2016). Regardless of splicing factor mutated status, chronic lymphocytic leukemia (CLL) and triple-negative breast cancer cells were highly sensitive to E7107, thereby identifying aberrant splicing as a targetable vulnerability in these tumor cells (Chan et al., 2017; Ten Hacken et al., 2018). Moreover, it was demonstrated that MYC drives hypersensitivity to spliceosome inhibition in various cancers, including breast cancers, suggesting a synthetic lethality relationship between oncogene activation and core spliceosome inhibition (Suda et al., 2017; Hepburn et al., 2018). These data indicated that splicing alterations could constitute one of the Achilles’ heels of cancer cells, thereby supporting the idea that cancer cells would be more sensitive to spliceosome inhibition than normal cells. More recently, the role of Bcl-xL and Mcl-1 as key determinants of cancer cell response to E7107 was shown in a large series of cancer cell lines, including NSCLC cells (Aird et al., 2019). Notably, it was found that the switch of Mcl-1 pre-mRNA splicing toward the pro-apoptotic short isoform Mcl-1_S_ is required for NSCLC cell death in response to E7107 treatment. These data revealed that modulation of the expression and/or of the ratio between pro- versus anti-apoptotic splice isoforms of several members of the Bcl-2 family controls cancer cell response to spliceosome inhibition. In addition, an orally available modulator of the SF3B complex, H3B-8800, was recently developed and was found to kill preferentially epithelial and hematologic tumor cells exhibiting mutations of spliceosome components (SF3B1, U2AF1, and SRSF2) (Seiler et al., 2018). H3B-8800 was granted orphan drug status by the FDA in August 2017 and is currently in clinical trials for the treatment of acute myelogenous leukemia and chronic myelomocytic leukemia. Other small-molecule inhibitors have been developed to target splicing factor kinases, such as the CDC2-like kinases (CLKs) and the serine-arginine protein kinases (SRPKs) (Lee and Abdel-Wahab, 2016; Lee et al., 2016). SRPIN340 blocks SRPK1-mediated phosphorylation of SRSF1, leading to splice switching of the pro-angiogenic VEGF_165_ to the anti-angiogenic VEGF_165_b splice variant of VEGF-A (Fukuhara et al., 2006; Siqueira et al., 2015). SPHINX31, a specific inhibitor of SRPK1, promoted the same splicing switch in favor of VEGF_165_b and inhibited tumor growth *in vivo* (Mavrou et al., 2015). Therefore, these compounds act as anti-angiogenic molecules through modulation of VEGF-A splicing, which appears to be crucial for tumor progression. Interestingly, SRPK1 was also recently identified as a cell-essential gene in acute myeloid leukemia (AML) cell lines driven by oncogenes derived from MLL fusion genes such as MLL-AF9 and MLL-AF6 (Tzelepis et al., 2016). The same group further demonstrated that SPHINX31 induces cell cycle arrest and leukemic cell differentiation and prolongs survival of mice transplanted with MLL-rearranged AML through the modulation of the splicing of many genes with established roles in leukemogenesis such as MYB, BRD4, and MED24 (Tzelepis et al., 2018). In particular, the authors demonstrated that the switch toward the long isoform of BRD4 induced by SRPK1 inhibition affects BRD4 recruitment to the chromatin in AML cells and is detected in breast cancer cells treated with SPHINX31 (Tzelepis et al., 2018). As SRPK1 and BRD4 proteins are involved in metastasis (Alsarraj et al., 2011; van Roosmalen et al., 2015), SPHINX31 may be used to prevent tumor dissemination. Pharmacological inhibition of the CLKs inhibited the phosphorylation of SRSF1, SRSF4, and SRSF6, reduced cell proliferation, and induced apoptosis notably through the regulation of S6K alternative splicing (Araki et al., 2015). Altogether, these studies highlighted the potential of targeting splicing alterations to slow down tumor progression, either through a global approach (i.e., spliceosome inhibition) or through the use of oligonucleotide-based therapies. They also identified Bcl-2 family members or Myc oncogene as critical determinants of tumor cell susceptibility to spliceosome inhibition.

### Alternative Splicing and Response to Therapies

As discussed, one of the major challenges in NSCLC patients today is to characterize the molecular mechanisms involved in the resistance to treatment in order to improve the duration of response and survival. Alterations in pre-mRNA alternative splicing have been reported during acquisition of drug resistance (Siegfried and Karni, 2018; Syafrizayanti et al., 2018; Wang and Lee, 2018). Below are some examples of splice variants that have been associated with the development of resistance in cancer (Table 1).

AR-v7, a constitutively activated splice variant of the androgen receptor (AR), was involved in disease progression and poor outcome upon AR-targeted therapies in castration-resistant prostate cancer patients (Antonarakis et al., 2017; Seitz et al., 2017). Accumulation of the FGFR2-IIIc splice variant of fibroblast growth factor receptor 2 (FGFR2) was associated with the progression of prostate cancer following androgen-based therapy (Sahadevan et al., 2007). In estrogen receptor-positive breast cancers, cyclin D1b, a splice variant of cyclin D1, correlated with the resistance to anti-estrogen therapy (Wang Y. et al., 2008). The BRCA1-delta11q splice variant, which lacks the majority of exon 11 of BRCA1 and consequently bypasses the inactivating germline mutations in this specific exon, promoted resistance to PARP inhibitors and cisplatin in breast cancers (Wang Y. et al., 2016). Pharmacological inhibition of the spliceosome reduced BRCA1-delta11q levels and sensitized cells harboring BRCA1 exon 11 mutations to PARP inhibitors. Aberrant splicing of BRCA2 was associated with resistance to mitomycin C (Meyer et al., 2017). In melanoma, acquired resistance to vemurafenib in patients with the BRAF(V600E) mutation was associated with the appearance of BRAF(V600E) splice variants such as those lacking exon cassette 4–8 (Poulikakos et al., 2011). Their specific decrease using splicing modulators limited the growth of vemurafenib-resistant cells or tumors (Salton et al., 2015). Other splice variants associated with acquisition of drug resistance include BCR-ABL35INS in chronic myelogenous leukemia (CML) treated with imatinib (Berman et al., 2016), a CD19 splice variant lacking exon 2 in B-cell acute lymphoblastic leukemia (B-ALLs) upon immunotherapy (Sotillo et al., 2015), and ER-alpha36, a splice variant of the estrogen receptor alpha, in ER-positive breast cancer patients upon tamoxifen therapy (Shi et al., 2009).

In lung cancer, a common deletion polymorphism in the BIM gene induces the preferential splicing of exon 3 over exon 4, leading to the synthesis of a BIM splice isoform lacking the BH3 domain (Ng et al., 2012). This polymorphism was associated with a shorter progression free survival in NSCLC patients treated with gefitinib, a tyrosine kinase inhibitor targeting epidermal growth factor receptor (EGFR-TKI) (Ng et al., 2012). Interestingly, vorinostat, an FDA-approved histone deacetylase inhibitor, corrected aberrant BIM splicing and counteracted EGFR-TKI resistance in NSCLC cell lines harboring the BIM deletion polymorphism (Nakagawa et al., 2013). Recently, we demonstrated that anti-angiogenic therapies targeting VEGFR1/VEGFR2 tyrosine kinase activity such as sunitinib, as well as bevacizumab (Avastin^R^), a monoclonal antibody against VEGF-A, increased the expression of both VEGF_165_b and sVEGFR1-i13 splice variants, which induced lung tumor escape to these therapies (Abou Faycal et al., 2018; Boudria et al., 2018). The alternative splicing of PTPMT1, a PTEN-like mitochondrial phosphatase, was also reported as a regulator of the response of lung cancer cells to radiation (sensitivity versus resistance) through the AMPK/mTOR signaling pathway (Sheng et al., 2018).

Immune checkpoint inhibitors (ICI) targeting the programmed cell death (PD-1)/programmed death ligand (PD-L1) pathways are used to counteract tumor immune escape in NSCLC patients (Meng et al., 2017). Although these treatments have radically changed the paradigm of care for these patients, a significant amount of them (9–29%) still progress, even hyper-progress, under treatment (Lo Russo et al., 2018). Up to now, the molecular mechanisms sustaining this worse response remain largely unknown. PD-1 displays alternative splice variants through differential exon skipping (Nielsen et al., 2005; Zhu and Lang, 2017). The PD-1 Deltaex3 splice variant does not have exon 3 and encodes a soluble isoform (sPD-1) (instead of a membrane-bound protein), which was not detectable in healthy individuals (Nielsen et al., 2005). Interestingly, increased sPD-1 levels were reported in NSCLC patients treated with erlotinib and were associated with prolonged progression-free and overall survival (Sorensen et al., 2016). Another PD-L1 splice variant that results from exon 2 skipping and encodes a soluble PD-L1 (sPD-L1), instead of membrane-bound PD-L1 (mPD-L1) isoform, was also described (He et al., 2005). In NSCLC patients, the expression of this circulating plasmatic sPD-L1 was up-regulated compared to the healthy controls and was associated with a bad prognosis (Cheng et al., 2015; Zhang et al., 2015; Okuma et al., 2017). As sPD-L1 may also be generated in response to mPD-L1 cleavage by metalloproteases, the contribution of alternative splicing in the biogenesis of sPD-L1 in lung cancer patients remains to be determined. Whether variations in the level of PD-1 or PD-L1 splice variants could account for distinct ICI response in lung cancer patients also remains to be tested.

As a whole, these data clearly define a link between dysregulated alternative splicing processing events and resistance to various therapies in cancer, including lung cancer. In this setting, it remains to be determined whether resistance could be reverted by using spliceosome inhibitors, either alone or in combination. In agreement with such a possibility, E7107 sensitized leukemic cells to venetoclax, a Bcl-2 inhibitor, by switching the splicing of Mcl-1 from Mcl-1_L_ (anti-apoptotic) to Mcl-1_S_ (pro-apoptotic) splice variant (Ten Hacken et al., 2018). The combination of SPHINX31 with an epigenetic drug, namely, i-BET-151, a BET inhibitor, showed synergistic effects against AML without noticeable toxicity in mice, suggesting the possible clinical use of SRPK1 inhibitors in combination with bromodomain inhibitors (Tzelepis et al., 2018). In addition, pladienolide B demonstrated a greater efficacy in lung cancer cell lines established from patients with prior chemotherapy (Suda et al., 2017) and significantly increased the sensitivity of various cancer cells to cisplatin (Anufrieva et al., 2018). Altogether, these studies highly support the idea that spliceosome inhibitors could be promising molecular target drugs in combination with conventional/targeted therapies.

### RNA Splice Variants: Predictive Biomarkers in Liquid Biopsies of Lung Cancer Patients?

As circRNAs, splice variants may be promising biomarkers for prognosis and post-treatment follow-up of cancer. Up to now, their detection has been done mainly in tumor tissue biopsies using either RNA sequencing and various PCR techniques for validation using primers allowing to distinguish between splice variants, or immunohistochemistry using specific antibodies of each splice variant. CTCs have also been used as a source to detect RNA splice variants. As an example, up-regulation of the splice variant delta5 of the estrogen receptor ESR1 was reported in CTCs isolated from whole blood of metastatic breast cancer patients compared to healthy blood donors (HBDs) (Beije et al., 2018). However, these analyses were complex because a subset of HBDs and healthy breast tissue also expressed this splice variant (Poola and Speirs, 2001). In contrast, full-length transcript and splice variants of the AR are typically absent in whole blood of HBDs (Onstenk et al., 2015). In metastatic prostate cancer, the expression of the AR splice variant V7 in CTCs was strongly associated with resistance to endocrine agents (Antonarakis et al., 2014, 2015). These results led to the development of a specific test to detect AR-V7 in CTCs from blood samples.

In contrast, the detection of circulating RNA splice variants in liquid biopsies (plasma, serum) is much more complex. Circulating free RNA degrades very quickly, and plasma samples need to be processed rapidly after blood extraction. The usual procedure is to freeze the sample at -80°C in an RNA preservative solution such as Trizol. In addition, the expression of splice variants by normal blood cells such as leukocytes may complicating the analyses. Nevertheless, some studies are promising. Seitz et al. (2017) showed that AR-v7 mRNA levels in whole blood of patients with castration-resistant prostate cancer could predict a poor outcome in response to androgen receptor-targeted therapies (abiraterone, enzalutamide). Somatic mutations affecting splice sites of exon 14 of the MET gene (METex14) have been reported in NSCLC (Ma et al., 2003; Kong-Beltran et al., 2006). These METex14 alterations induced constitutive activation of MET kinase activity (Frampton et al., 2015), and MET-targeted therapies are now used in MET14-positive NSCLC patients (Onozato et al., 2009; Paik et al., 2015). Recently, METex14 splice variant was detected in platelet-derived RNA from a NSCLC patient who partially responded to crizotinib (Aguado et al., 2016). Although this result has to be confirmed, this suggests that detection of METex14 splice variants in liquid biopsies could help to select good responders to MET-targeted therapies.

As a whole, it is clear that the detection of RNA splice variants in liquid biopsies, despite remaining a challenge, is a promising field.

## Conclusion and Perspectives

There is a crucial need of both sensitive and specific cancer biomarkers, including proteins, DNAs, or RNAs, for early diagnosis, prognosis, and patient follow-up. Tissue biopsies have been widely used as a reservoir of these biomarkers. However, tissue biopsies are invasive procedures and some patients are not eligible, especially for repeated collection. In addition, tissue biopsies do not mirror tumor heterogeneity and metastatic burden of most advanced tumors. In contrast, liquid biopsies are less invasive than tissue biopsies, and circulating biomarkers more likely reflect intratumoral heterogeneity and dissemination. However, we are facing other challenges mostly involving the way to collect body fluids, the choice of biological materials to be analyzed (saliva, urine, whole blood, circulating cells, and platelet-enriched or platelet-deprived plasma), the selection of appropriate controls, and the standardization and optimization of pre-analytical and analytical procedures. It is clear that improvement of all these steps will be required in the future if we want to use liquid biopsies as a surrogate of tissue biopsies to detect and quantify accurate cancer biomarkers.

We have to select the most accurate genome-wide transcriptomic analyses to profile circRNAs and RNA splice variants in tumor patients. In this setting, we have to consider the reproducibility, the sensitivity, and the cost of these analyses if we want to translate them into the clinic. Deep RNA-sequencing allows the identification of previously known as well as unknown circRNAs and RNA splice variants, and sensitivity can be improved by increasing sequencing depth, which may also increase the cost. RNA-seq allows a better determination of gene/transcript concentration. However, for circRNAs, the detection efficiency is limited, as only 0.1% reads from RNA-seq cover the head-to-tail junction (Szabo and Salzman, 2016). Li S. et al. (2018b) recently compared the circRNA detection efficiency of RNA-Seq (20 million sequencing reads) and a circRNA microarray containing probes targeting back-splice site of 87,935 circRNA sequences covering most of the circRNAs represented in circBase. The results showed that the microarray is more efficient than RNA-Seq for circRNA profiling. CircRNA microarray detected about 80,000 circRNAs in matched cervical tumors and normal tissues from 10 patients with about 25,000 circRNAs differentially expressed in tumors. Around 18,000 circRNAs were also detected in cell-free plasma samples of 21 patients with cervical cancer, and approximately 2,700 of them were differentially expressed after surgical tumor removal (Li S. et al., 2018b). Therefore, besides RNA-Seq, these data suggested that circRNA microarrays could be an efficient and sensitive tool for the profiling of specific circRNAs in tumor liquid biopsies. Moreover, Romero et al. (2018) compared the performance of RNA-Seq and splice junction arrays for the analysis of transcript splicing events in breast cancer cell lines treated with a drug that affects splicing. They observed a high degree of coherence between the two technologies, with the detection power of the junction array being equivalent to RNA-Seq with up to 60 million reads. Nazarov et al. (2017) compared RNA sequencing (Illumina 2000 RNA-seq) and transcriptome arrays (Affymetrix HTA 2.0) in patient samples derived from normal lung epithelium and squamous cell lung carcinoma. The analysis of alternative splicing, more specifically differential exon usage, produced discordant results between the two platforms, with higher stochastic variability and insufficient reads for many exons and exon junctions in RNA-Seq. Therefore, microarray techniques could also constitute a faster and cheaper alternative to RNA-sequencing for RNA splice variant profiling, especially to compare relatively large groups of samples or to focus on well-annotated transcriptional regions and already known RNA splice variants.

The interest of large-scale RNA analyses in body fluids is to establish the landscape of cell-free RNA transcriptome, to provide new insights into the temporal dynamics of circulating RNAs, and to estimate the relative contribution of tissues to the pool of circulating RNAs through the analyses of tissue-specific genes. In the RNA field, RNA splice variants and circRNAs have recently emerged as cancer biomarker candidates, mainly because thousands of them are aberrantly expressed in various cancer types compared to normal tissues. As a consequence, many papers have proposed specific RNA splice variants or circRNAs as cancer biomarkers or as useful tools for prediction of patients’ outcome. However, in most of the cases, the biological consequences of their deregulation remain unknown. In addition, the vast majority of circRNA biomarker studies published to date are retrospective. It is therefore hard to decipher whether these circRNAs and RNA splice variants belong to a common driving mechanism of oncogenesis or are just common by-product/end-product of oncogenesis. Very recently, using an ultra-deep non-poly-A RNA-seq on 144 localized prostate tumors, Chen et al. (2019) showed that the abundance of both global and specific circRNAs is associated with clinical outcome. These results suggested that some circRNAs play oncogenic roles. As a whole, these data highlight the need to characterize the functions of these aberrantly expressed RNA splice variants or circRNAs in order to understand their contribution to tumor progression and/or tumor escape to therapies. Moreover, as RNA splice variants or circRNAs could be highly expressed in some blood cells such as platelets, their detection in whole blood could only reflect the level of circulating cells. Therefore, a careful comparison of their expression level between healthy donors and cancerous patients, a thorough description of serum/plasma transportation mechanisms, and the study of the origin of these RNA splice variants or circRNAs with accurate methods should be considered as preliminary steps before proposing them as relevant cancer biomarkers. Keeping these precautions in mind, it is likely that some of these circulating RNAs will emerge as relevant cancer biomarkers in the near future and enter into clinical practice.

## Author Contributions

FdF, SG, WC, and BE wrote the manuscript. All authors read and approved the manuscript.

## Conflict of Interest Statement

The authors declare that the research was conducted in the absence of any commercial or financial relationships that could be construed as a potential conflict of interest.
