# Supplementary material for: Circular RNAs and RNA Splice Variants as Biomarkers for Prognosis and Therapeutic Response in the Liquid Biopsies of Lung Cancer Patients
Source: Front Genet. 2019 May 7;10:390. doi: 10.3389/fgene.2019.00390 (PMC6514155; doi:10.3389/fgene.2019.00390)
Supplement: Supplementary file 1 [file Table_1.docx]

**Supplementary Table 1:** Examples of circRNAs (alias are also indicated) found to be up-regulated in lung tumors compared to normal lung together with their corresponding gene symbol (in alphabetical order). Ding and colleagues performed RNA sequencing in 3 lung adenocarcinoma patients and 3 normal lung tissues (Ding et al., 2018). Qiu and colleagues performed comparison between 5 lung adenocarcinoma and 5 normal lung tissues (Qiu M et al., 2018). Some of these circRNAs (in bold and italic) were also detected in the A549 lung adenocarcinoma cell line.

| **CircRNA name** | **Alias** | **Gene Symbol** |  |  | **References** |
| --- | --- | --- | --- | --- | --- |
| hsa-circRNA8859-7 | hsa_circ_0009175 | **ACBD6** |  |  | Ding et al |
| hsa_circRNA_103602 | hsa_circ_0069152 | **AFAP1** |  |  | Qiu et al |
| hsa-circRNA9918-7 | hsa_circ_0003770 | **ARF3** |  |  | Ding et al |
| hsa-circRNA9097-12 | hsa_circ_0000231 | ***ARHGAP12*** |  |  | Ding et al |
| hsa-circRNA1579-15 | hsa_circ_0024627 | ***ARHGEF12*** |  |  | Ding et al |
| hsa_circRNA_101900 | hsa_circ_0040809 | **BANP** |  |  | Qiu et al |
| hsa_circRNA_103243 | hsa_circ_0001238 | ***CCDC134*** |  |  | Qiu et al |
| hsa_circRNA_104499 | hsa_circ_0082564 | ***CREB3L2*** |  |  | Qiu et al |
| hsa-circRNA952-5 | hsa_circ_0008865 | ***DDX21*** |  |  | Ding et al |
| hsa-circRNA851-55 | hsa_circ_0007813 | ***DHTKD1*** |  |  | Ding et al |
| hsa_circRNA_104545 | hsa_circ_0002451 | **DNAJB6** |  |  | Qiu et al |
| hsa-circRNA7559-13 |  | ***EFR3A*** |  |  | Ding et al |
| hsa_circRNA_100833 | hsa_circ_0022383 | ***FADS2*** |  |  | Qiu et al |
| hsa-circRNA837-10 | hsa_circ_0000209 | **FAM208B** |  |  | Ding et al |
| hsa_circRNA_103511 | hsa_circ_0067971 | **FNDC3B** |  |  | Qiu et al |
| hsa_circRNA_103512 | hsa_circ_0001361 | **FNDC3B** |  |  | Qiu et al |
| hsa_circRNA_103513 | hsa_circ_0006948 | **FNDC3B** |  |  | Qiu et al |
| hsa_circRNA_101002 | hsa_circ_0025201 | **GAPDH** |  |  | Qiu et al |
| hsa-circRNA15922-3 | hsa_circ_0002883 | **GOLGA1** |  |  | Ding et al |
| hsa-circRNA15137-28 |  | **GRB10** |  |  | Ding et al |
| hsa-circRNA13699-6 | hsa_circ_0066959 | **HCLS1** |  |  | Ding et al |
| hsa_circRNA_104336 | hsa_circ_0003958 | ***HIBADH*** |  |  | Qiu et al |
| hsa-circRNA15822-56 | hsa_circ_0006702 | **IARS** |  |  | Ding et al |
| hsa-circRNA9793-1 | hsa_circ_0024948 | **KDM5A** |  |  | Ding et al |
| hsa-circRNA471-11 | hsa_circ_0000118 | **MAN1A2** |  |  | Ding et al |
| hsa-circRNA471-26 | hsa_circ_0000119 | **MAN1A2** |  |  | Ding et al |
| hsa-circRNA14085-4 | hsa_circ_0001432 | ***MANBA*** |  |  | Ding et al |
| hsa-circRNA978-6 | hsa_circ_0000247 | **MCU** |  |  | Ding et al |
| hsa-circRNA7013-1 |  | **MPP6** |  |  | Ding et al |
| hsa-circRNA7013-28 | hsa_circ_0001686 | **MPP6** |  |  | Ding et al |
| hsa_circRNA_102231 | hsa_circ_0046263 | ***P4HB*** |  |  | Qiu et al |
| hsa-circRNA1090-50 | hsa_circ_0000258 | **PDCD11** |  |  | Ding et al |
| hsa_circRNA_103141 | hsa_circ_0008021 | ***PDXK*** |  |  | Qiu et al |
| hsa-circRNA5171-3 | hsa_circ_0008021 | ***PDXK*** |  |  | Ding et al |
| hsa-circRNA8492-5 | hsa_circ_0012300 | **PIK3R3** |  |  | Ding et al |
| hsa_circRNA_103510 | hsa_circ_0067934 | **PRKCI** |  |  | Qiu et al |
| hsa_circRNA_101085 | hsa_circ_0027089 | **PTGES3** |  |  | Qiu et al |
| hsa-circRNA6529-19 | hsa_circ_0001551 | **RARS** |  |  | Ding et al |
| hsa-circRNA4138-4 | hsa_circ_0008590 | **RELB** |  |  | Ding et al |
| hsa-circRNA5772-22 | hsa_circ_0001346 | **RNF13** |  |  | Ding et al |
| hsa-circRNA5772-28 | hsa_circ_0003956 | **RNF13** |  |  | Ding et al |
| hsa_circRNA_100156 | hsa_circ_0000048 | ***RNF19B*** |  |  | Qiu et al |
| hsa_circRNA_103123 | hsa_circ_0002360 | ***RUNX1*** |  |  | Qiu et al |
| hsa_circRNA_104119 | hsa_circ_0076691 | **RUNX2** |  |  | Qiu et al |
| hsa_circRNA_103348 | hsa_circ_0065214 | **SCAP** |  |  | Qiu et al |
| hsa-circRNA13515-32 | hsa_circ_0007291 | **SCAP** |  |  | Ding et al |
| hsa-circRNA8327-6 |  | ***SDHB*** |  |  | Ding et al |
| hsa_circRNA_100539 | hsa_circ_0017627 | **SFMBT2** |  |  | Qiu et al |
| hsa_circRNA_100543 | hsa_circ_0000211 | **SFMBT2** |  |  | Qiu et al |
| hsa_circRNA_104018 | hsa_circ_0005714 | ***SFXN1*** |  |  | Qiu et al |
| hsa-circRNA7203-6 |  | **SH2B2** |  |  | Ding et al |
| hsa_circRNA_103987 | hsa_circ_0004104 | **SPARC** |  |  | Qiu et al |
| hsa-circRNA7444-39 |  | **SPIDR** |  |  | Ding et al |
| hsa-circRNA158-7 | hsa_circ_0008126 | **STX12** |  |  | Ding et al |
| hsa-circRNA4084-20 | hsa_circ_0000932 | **SUPT5H** |  |  | Ding et al |
| hsa-circRNA3327-39 | hsa_circ_0006220 | **TADA2A** |  |  | Ding et al |
| hsa-circRNA10148-13 | hsa_circ_0000446 | **TAOK3** |  |  | Ding et al |
| hsa_circRNA_102254 | hsa_circ_0046522 | **TBCD** |  |  | Qiu et al |
| hsa_circRNA_102259 | hsa_circ_0046533 | **TBCD** |  |  | Qiu et al |
| hsa-circRNA398-1 |  | **TMEM56** |  |  | Ding et al |
| hsa-circRNA13828-14 | hsa_circ_0067946 | ***TNIK*** |  |  | Ding et al |
| hsa-circRNA8986-20 | hsa_circ_0000195 | **TTC13** |  |  | Ding et al |
| hsa-circRNA12521-94 | hsa_circ_0001016 | **XPO1** |  |  | Ding et al |
| hsa_circRNA_102288 | hsa_circ_0046702 | **YES1** |  |  | Qiu et al |
| hsa_circRNA_104667 | hsa_circ_0005962 | ***YWHAZ*** |  |  | Qiu et al |
| hsa-circRNA11177-9 | hsa_circ_0007963 | **ZFHX3** |  |  | Ding et al |
| hsa_circRNA_103809 | hsa_circ_0072088 | **ZFR** |  |  | Qiu et al |
| hsa-circRNA9019-2 | hsa_circ_0017348 | ***ZNF124*** |  |  | Ding et al |
