# Supplementary material for: Circular RNAs and RNA Splice Variants as Biomarkers for Prognosis and Therapeutic Response in the Liquid Biopsies of Lung Cancer Patients
Source: Front Genet. 2019 May 7;10:390. doi: 10.3389/fgene.2019.00390 (PMC6514155; doi:10.3389/fgene.2019.00390)
Supplement: Supplementary file 2 [file Table_2.docx]

**Supplementary Table 2:** Examples of circRNAs (alias are also indicated) found to be down-regulated in lung tumors compared to normal lung together with their corresponding gene symbols (in alphabetical order). Ding and colleagues performed RNA sequencing in 3 lung adenocarcinoma patients and 3 normal lung tissues (Ding et al., 2018). Qiu and colleagues performed comparison between 5 lung adenocarcinoma and 5 normal lung tissues (Qiu M et al., 2018).

| **CircRNA name** | **Alias** | **Gene Symbol** | **References** |
| --- | --- | --- | --- |
| hsa_circRNA_101282 | hsa_circ_0030569 | **ABCC4** | Qiu et al |
| hsa_circRNA_102045 | hsa_circ_0002404 | **ACACA** | Qiu et al |
| hsa_circRNA_101136 | hsa_circ_0028123 | **ACACB** | Qiu et al |
| hsa_circRNA_000926 | hsa_circ_0001022 | **ACTR2** | Qiu et al |
| hsa_circRNA_101773 | hsa_circ_0004260 | **ARHGAP17** | Qiu et al |
| hsa_circRNA_000274 | hsa_circ_0000919 | **ATP13A1** | Qiu et al |
| hsa_circRNA_000864 | hsa_circ_0000662 | **AXIN1** | Qiu et al |
| hsa_circRNA_104342 | hsa_circ_0003162 | **BBS9** | Qiu et al |
| hsa_circRNA_000963 | hsa_circ_0001644 | **BCLAF1** | Qiu et al |
| hsa_circRNA_001350 | hsa_circ_0000253 | **BLNK** | Qiu et al |
| hsa_circRNA_104736 | hsa_circ_0086414 | **BNC2** | Qiu et al |
| hsa_circRNA_105013 | hsa_circ_0001936 | **BRWD3** | Qiu et al |
| hsa_circRNA_100660 | hsa_circ_0019390 | **C10orf28** | Qiu et al |
| hsa_circRNA_103137 | hsa_circ_0061817 | **C2CD2** | Qiu et al |
| hsa_circRNA_104858 | hsa_circ_0087897 | **C9orf5** | Qiu et al |
| hsa-circRNA15984-3 | hsa_circ_0001900 | **CAMSAP1** | Ding et al |
| hsa_circRNA_102774 | hsa_circ_0055412 | **CAPG** | Qiu et al |
| hsa_circRNA_102446 | hsa_circ_0049356 | **CARM1** | Qiu et al |
| hsa-circRNA10960-39 | hsa_circ_0007637 | **CREBBP** | Ding et al |
| hsa_circRNA_103749 | hsa_circ_0005480 | **DCLK2** | Qiu et al |
| hsa-circRNA9727-19 | hsa_circ_0004293 | **DDX6** | Ding et al |
| hsa_circRNA_100719 | hsa_circ_0020390 | **DOCK1** | Qiu et al |
| hsa_circRNA_100832 | hsa_circ_0022378 | **FADS1** | Qiu et al |
| hsa_circRNA_000104 | hsa_circ_0000266 | **FAM53B** | Qiu et al |
| hsa_circRNA_103249 | hsa_circ_0063756 | **FBLN1** | Qiu et al |
| hsa_circRNA_400036 | hsa_circ_0092284 | **FDXR** | Qiu et al |
| hsa_circRNA_100988 | hsa_circ_0000369 | **FLI1** | Qiu et al |
| hsa_circRNA_100989 | hsa_circ_0000370 | **FLI1** | Qiu et al |
| hsa_circRNA_103674 | hsa_circ_0070113 | **FRAS1** | Qiu et al |
| hsa_circRNA_104126 | hsa_circ_0076798 | **GCLC** | Qiu et al |
| hsa-circRNA2123-2 | hsa_circ_0007259 | **GTF2F2** | Ding et al |
| hsa_circRNA_104400 | hsa_circ_0006944 | **GTF2I** | Qiu et al |
| hsa_circRNA_104401 | hsa_circ_0005513 | **GTF2I** | Qiu et al |
| hsa_circRNA_000911 | hsa_circ_0001184 | **IFNGR2** | Qiu et al |
| hsa-circRNA2744-21 | hsa_circ_0005035 | **IGF1R** | Ding et al |
| hsa_circRNA_103733 | hsa_circ_0003390 | **INTU** | Qiu et al |
| hsa_circRNA_102442 | hsa_circ_0049271 | **KEAP1** | Qiu et al |
| hsa_circRNA_100395 | hsa_circ_0015278 | **KLHL20** | Qiu et al |
| hsa_circRNA_102450 | hsa_circ_0006877 | **LDLR** | Qiu et al |
| hsa_circRNA_001288 | hsa_circ_0000999 | **LOC100506142** | Qiu et al |
| hsa_circRNA_100302 | hsa_circ_0013587 | **LRIG2** | Qiu et al |
| hsa_circRNA_102551 | hsa_circ_0003859 | **LTBP4** | Qiu et al |
| hsa_circRNA_104200 | hsa_circ_0007798 | **MAP3K5** | Qiu et al |
| hsa-circRNA68-17 | hsa_circ_0006470 | **MFN2** | Ding et al |
| hsa_circRNA_100499 | hsa_circ_0004039 | **MTR** | Qiu et al |
| hsa_circRNA_101278 | hsa_circ_0004217 | **MYCBP2** | Qiu et al |
| hsa_circRNA_104513 | hsa_circ_0007518 | **NDUFB2** | Qiu et al |
| hsa_circRNA_101969 | hsa_circ_0041821 | **NEURL4** | Qiu et al |
| hsa-circRNA6258-29 | hsa_circ_0005585 | **NNT** | Ding et al |
| hsa_circRNA_102222 | hsa_circ_0046178 | **NPLOC4** | Qiu et al |
| hsa_circRNA_100850 | hsa_circ_0006857 | **PACS1** | Qiu et al |
| hsa-circRNA13375-6 | hsa_circ_0002610 | **PACSIN2** | Ding et al |
| hsa_circRNA_100891 | hsa_circ_0023685 | **PAK1** | Qiu et al |
| hsa_circRNA_101712 | hsa_circ_0005627 | **PARN** | Qiu et al |
| hsa-circRNA5198-38 | hsa_circ_0002903 | **PCNT** | Ding et al |
| hsa_circRNA_104791 | hsa_circ_0087234 | **PCSK5** | Qiu et al |
| hsa_circRNA_103727 | hsa_circ_0008144 | **PDE5A** | Qiu et al |
| hsa_circRNA_102600 | hsa_circ_0000958 | **PPP1R12C** | Qiu et al |
| hsa_circRNA_100136 | hsa_circ_0000045 | **PUM1** | Qiu et al |
| hsa_circRNA_103428 | hsa_circ_0004639 | **PVRL3** | Qiu et al |
| hsa_circRNA_103271 | hsa_circ_0064019 | **RABL2B** | Qiu et al |
| hsa_circRNA_103377 | hsa_circ_0001304 | **RBM5** | Qiu et al |
| hsa_circRNA_101373 | hsa_circ_0005139 | **RDH11** | Qiu et al |
| hsa_circRNA_400033 | hsa_circ_0092360 | **RPL23A** | Qiu et al |
| hsa-circRNA8462-8 | hsa_circ_0000061 | **SCMH1** | Ding et al |
| hsa_circRNA_104099 | hsa_circ_0076092 | **SCUBE3** | Qiu et al |
| hsa_circRNA_100608 | hsa_circ_0002624 | **SLC25A16** | Qiu et al |
| hsa_circRNA_101737 | hsa_circ_0006434 | **SMG1** | Qiu et al |
| hsa_circRNA_102171 | hsa_circ_0004789 | **SMURF2** | Qiu et al |
| hsa_circRNA_100748 | hsa_circ_0020926 | **STIM1** | Qiu et al |
| hsa_circRNA_000993 | hsa_circ_0001887 | **STRBP** | Qiu et al |
| hsa_circRNA_103558 | hsa_circ_0068641 | **TM4SF19** | Qiu et al |
| hsa_circRNA_101308 | hsa_circ_0031027 | **TMCO3** | Qiu et al |
| hsa_circRNA_104551 | hsa_circ_0083294 | **TNKS** | Qiu et al |
| hsa_circRNA_104121 | hsa_circ_0076767 | **TRAM2** | Qiu et al |
| hsa_circRNA_001800 | hsa_circ_0001033 | **TTC31** | Qiu et al |
| hsa_circRNA_103211 | hsa_circ_0063158 | **TXN2** | Qiu et al |
| hsa_circRNA_000942 | hsa_circ_0001303 | **UBA7** | Qiu et al |
| hsa_circRNA_101957 | hsa_circ_0003239 | **UBE2G1** | Qiu et al |
| hsa_circRNA_101958 | hsa_circ_0041555 | **UBE2G1** | Qiu et al |
| hsa_circRNA_104803 | hsa_circ_0087354 | **UBQLN1** | Qiu et al |
| hsa_circRNA_102728 | hsa_circ_0006110 | **USP34** | Qiu et al |
| hsa_circRNA_102741 | hsa_circ_0003497 | **WDPCP** | Qiu et al |
| hsa_circRNA_001587 | hsa_circ_0000979 | **XLOC_001374** | Qiu et al |
| hsa_circRNA_101706 | hsa_circ_0005394 | **ZC3H7A** | Qiu et al |
| hsa_circRNA_104310 | hsa_circ_0079385 | **ZDHHC4** | Qiu et al |
| hsa_circRNA_100160 | hsa_circ_0011536 | **ZMYM4** | Qiu et al |
| hsa_circRNA_102602 | hsa_circ_0052318 | **ZNF418** | Qiu et al |
